# 

**Published:** 1980

**Authors:** 


### Contents

3 The Medical Adviser and General Practitioner Trials
R. P. Bax

7 Dermatological do-it-yourself
J. L. Burton

9 Imitation
Philip Radford

11 Abstracts

